# Notes on New Drugs and Preparations for the Sick

**Published:** 1902-09

**Authors:** 


### Notes on New Drugs and Preparations for the Sick.

---

**Glyco-Heroin; Ergoapiol.**—MARTIN H. SMITH CO., New York; CHRISTY & CO., London.—We have received samples of these proprietary preparations of well-known drugs.

The Heroin compound is a glycerine solution containing one-sixteenth of a grain of heroin in each teaspoonful dose, with the addition of white pine bark, ammonium hypophosphite, and balsam of tolu. It forms a dark amber liquid, clear, with an agreeable aromatic odour and taste. Heroin is rapidly becoming established as a useful sedative in respiratory conditions where a cough sedative is desirable, and it appears to have many advantages over morphia, codeia, and other opium alkaloids. It rarely fails to give marked relief, even when given alone in tabloid or powder, but it seems likely that such a combination as this will be more effective than the simple solution of heroin.

The Ergoapiol capsules have given excellent results as an ideal hæmagogue and emmenagogue combination.

---

**Cascara Evacuant.**—PARKE, DAVIS & CO., London.—Yet another preparation of Cascara. It is difficult to say which is the best. This one is described as "an active and agreeable preparation of the unchanged bitterless glucosides of cascara sagrada, possessing all the desirable laxative properties of this valuable drug." It is based upon the discovery that the laxative effect is due not alone to the presence of the bitter glucoside, but also to the action of a bitterless glucoside. It is prepared without the use of alkalies, which commonly destroy some portion of the bitterless laxative, and in a concentrated form, the dose ranging from 10 to 30 minims. It has a pleasant aromatic taste, absolutely free from the intense bitter of the ordinary liquid extract of the rhamnus purshianus.

---

**Enzymol.**—FAIRCHILD BROS. & FOSTER.—A purified solution of the proteolytic enzyme, especially prepared for external application. It is supposed to dissolve all septic matter, correct offensive odours, and impart a healthy stimulus to the affected surface. By the addition of an equal volume of water it becomes an artificial gastric juice, which may be applied freely to the surface of wounds either by spray, compress, or injection.

**Tabloids and Soloids.**—BURROUGHS, WELLCOME & Co., London.

**Kino Compound Powder**, B.P. gr. 5.—Kino is a well known, but little used, astringent, of considerable service in the treatment of certain forms of diarrhœa and dysentery. This five grain tabloid is a convenient form for its administration. It will be remembered that this compound powder contains 5 per cent. of opium.

**Compound Liquorice Powder**, gr. 30.—It is a common occurrence for patients to refuse to take compound liquorice powder as ordinarily prescribed, on account of its unpleasant taste when mixed with water. The "Tabloid" has been introduced with the view of providing a convenient means of administering this well-known laxative. Each represents thirty grains of the Compound Liquorice Powder of the British Pharmacopœia, and two to four may be swallowed, with water, for a dose.

**Ammonium Chloride and Liquorice.**—

Ammon. Chloride, gr. 3.
Ext. Glycyrrhizæ, gr. ij.

It has been long recognised that the unpleasant taste of ammonium chloride may be disguised by the addition of extract of liquorice. Each "Tabloid" product contains three grains of ammonium chloride and two grains of extract of liquorice, and presents an extremely convenient means of administering the combination. One may be allowed to dissolve slowly in the mouth as often as may be necessary.

**Apomorphine Compound.**—

| | | |
|---|---|---|
| Apomorphine Hydrochloride, | gr. 1/50 | (0.0013 gm.) |
| Ammonium Chloride ... ... | gr. 3 | (0.194 gm.) |
| Extract of Liquorice ... ... | gr. 1½ | (0.097 gm.) |

As is well known, apomorphine, in small doses, frequently repeated, is used internally in the treatment of certain affections of the respiratory organs; and it is said to be of especial value in cases of chronic bronchitis, bronchorrhœa and whooping cough. A useful combination is presented in "Tabloid" Apomorphine Compound, in which product the action of the apomorphine is aided by the stimulant-expectorant properties of ammonium chloride and by the demulcent qualities of extract of liquorice. One may be slowly dissolved in the mouth, or swallowed with a little water, every hour or every other hour, as the symptoms of the patient may indicate.

**Soloid Sodium Hydroxide**, gr. 2;
**Soloid Pyrogallic Acid**, gr. ½.

These products have recently been introduced with the view of providing a ready means of estimating the degree of oxygenation of a sewage effluent. It is generally admitted that a sewage effluent which is well charged with oxygen will almost certainly be satisfactory, whereas if the effluent contains little or no free oxygen it will be unsatisfactory. The examination by means of the above "Soloid" products is very simple, and may be conducted in the following manner:—Fill a wide-mouthed bottle, holding about four ounces, with the effluent as it runs from the beds, drop in one "Soloid" product of each reagent and insert the stopper carefully and in such a way as not to include any bubble of air. Shake the bottle for about half a minute and note the effect produced. If the liquid remain colourless or only faintly coloured, the oxygenation is not satisfactory. If the liquid assume a rich red-brown colour, the oxygenation is satisfactory. We have made experiments with these and find they do exactly what is claimed for them.

